# Supplementary material for: Intracellular trafficking of furin enhances cellular intoxication by recombinant immunotoxins based on Pseudomonas exotoxin A
Source: Biol Open. 2025 Jun 20;14(6):bio061792. doi: 10.1242/bio.061792 (PMC12208400; doi:10.1242/bio.061792)
Supplement: Supplementary information [file biolopen-14-061792-s1.pdf]

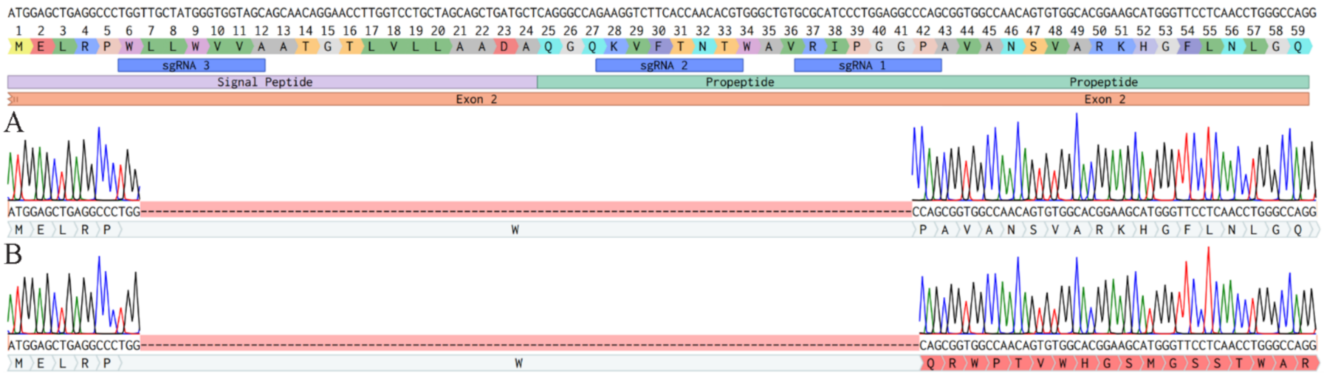

Fig. S1. ΔFur293 cells contain a deletion in exon 2 of the furin gene. Alignment of exon 2 from human furin with sequencing results from ΔFur293. Panel A shows a representative chromatogram of the 105 bp deletion. Panel B shows a representative chromatogram of the 106 bp deletion. Images were generated using Benchling[1].

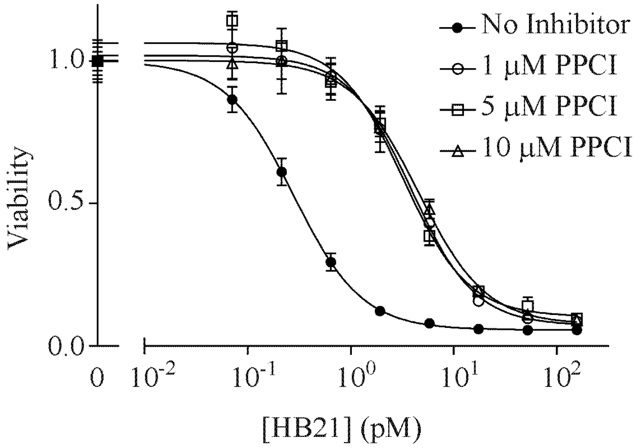

Fig. S2. PPCI diminishes the toxicity of HB21. The cytotoxicity of HB21 was evaluated against HEK293 FRT cells in the presence of increasing concentrations of proprotein convertase inhibitor (PPCI). Error bars indicate the standard error of the mean for a minimum of 5 replicates at each toxin concentration. (No Inhibitor EC_{50} = 0.2806 pM; 1 µM PPCI EC_{50} = 3.923 pM; 5 µM PPCI EC_{50} = 3.263 pM; 10 µM PPCI EC_{50} = 4.557 pM).

Biology Open • Supplementary information

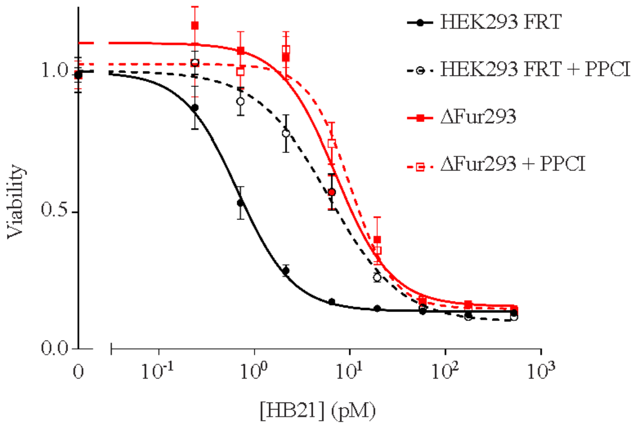

Fig. S3. Example HB21 cytotoxicity assays. The cytotoxicity of HB21 was evaluated against HEK293 FRT and ΔFur293 cells in the absence and presence of1 μM PPCI. Errorbars denote standard error values for a minimum of 5 replicates at each toxin concentration.

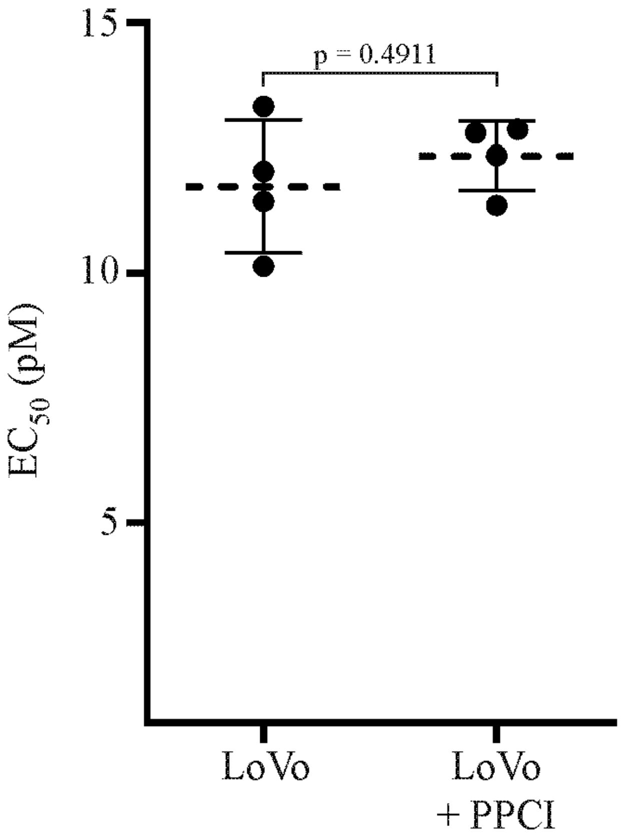

Fig. S4. Cytotoxicity evaluation of PPCI-treated LoVo cells. Furin-deficient LoVo cells were treated with HB21 in the presence and absence of 1 μM PPCI.EC_{50} (pM) values from four separate paired assays are plotted. Dotted lines denote the average value for each condition and error bars indicate the standard deviation. No significant difference was detected using a T-test performed as described.

Biology Open • Supplementary information

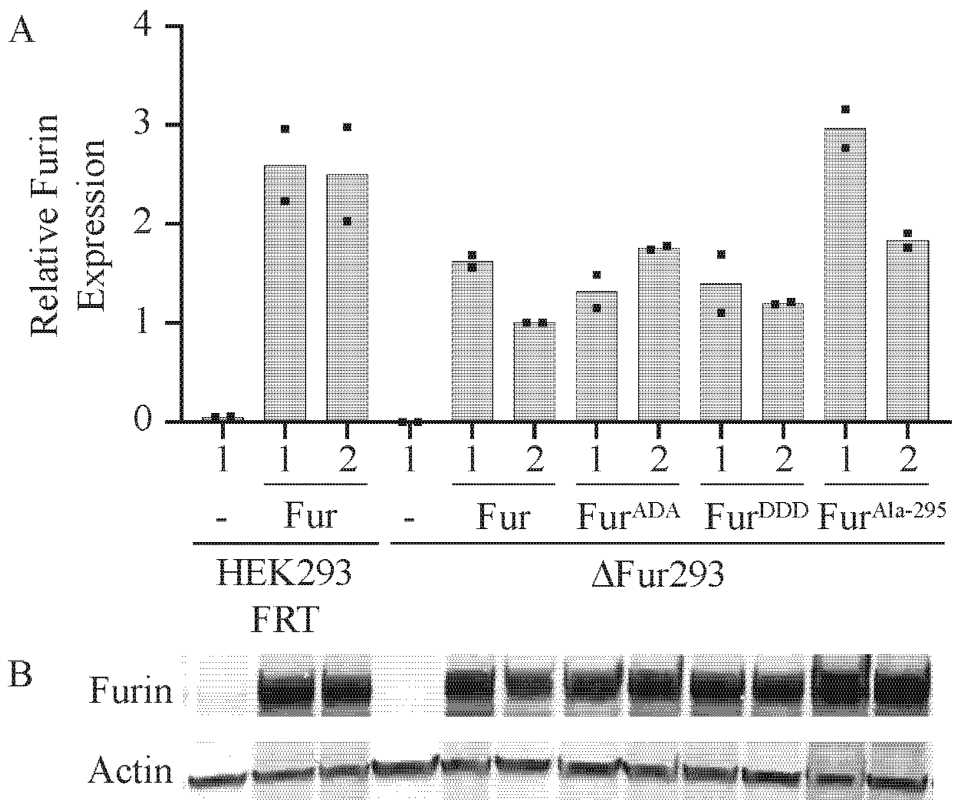

Fig. S5. Furin expression levels. Cell lysates from HEK293 FRT and ΔFur293 cell lines with and without transgenic furin (Fur, Fur^{ADA}, Fur^{DDD}, or Fur$^{Ala\text{-}295}$) were evaluated for furin expression by western blot. Two separate clones from each transgenic line were evaluated, and each line was evaluated twice. One representative western blot (B) is shown. Band intensity from each western blot was calculated and normalized. Results are plotted as the average of two experiments (A). Normalized expression values were used to correct for differences in response to HB21-LR cytotoxicity.

Biology Open • Supplementary information

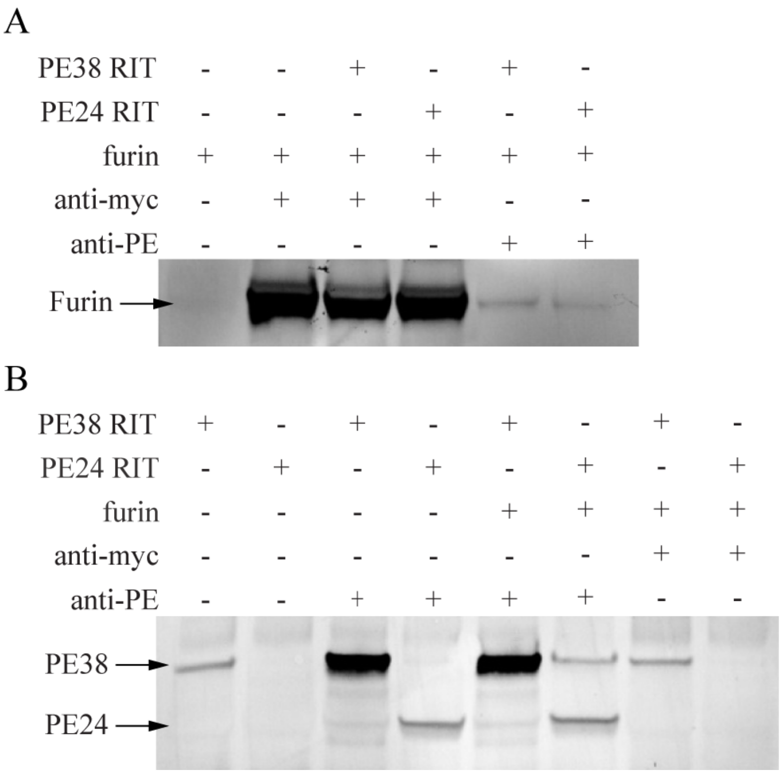

Fig. S6. In vitro co-immunoprecipitation of furin and PE-based RITs. Co-immunoprecipitation of myc/FLAG-tagged furin incubated with HB21 (PE38 RIT) or HB21-LR (PE24 RIT). Panel A shows a blot probed with anti-FLAG mouse mAb. Proteins included in each condition are indicated above each lane. Arrow indicates the position of furin. Panel B shows a blot probed with anti-PE polyclonal antibody. Proteins included in each condition are indicated above each lane. Arrows indicate the positions of HB21 (PE38) and HB21-LR (PE24).

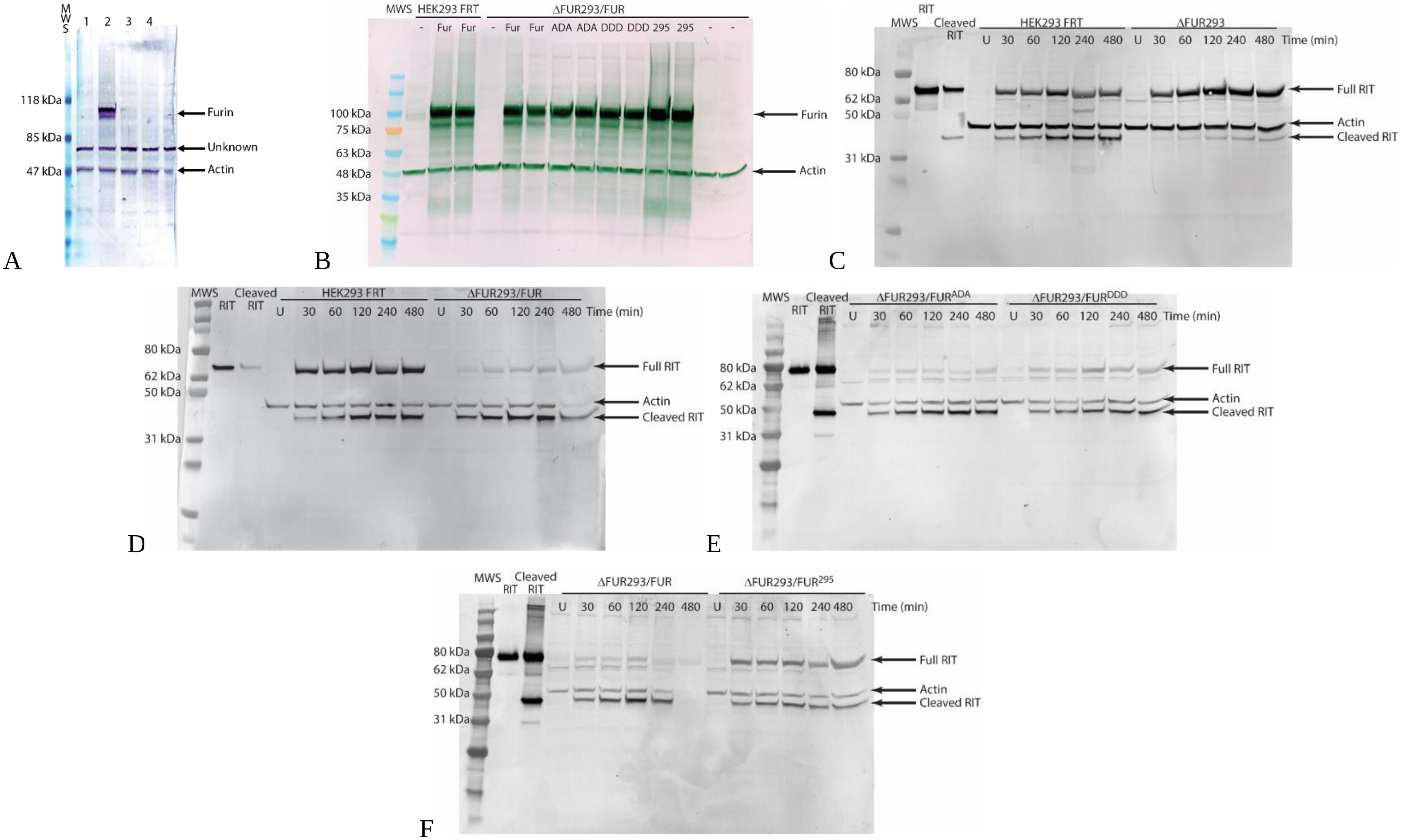

Fig. S7. Example western blots. Scanned images of representative original western blots to detect furin from Figure 1 (A) and Figure S6 (B), and to detect PE in Figure 4 (C & D) and Figure 6 (E & F).

Table S1. Oligonucleotides

Oligonucleotide	Sequence
Furin CRISPR sgRNA1	5'GCG CAT CCC TGG AGG CCC AG
Furin CRISPR sgRNA2	5'GAA GGT CTT CAC CAA CAC GT
Furin CRISPR sgRNA3	5'GCT ACC ACC CAT AGC AAC CA
pCMV6 Insert Start For	5'TCT AGT AAG CTT GCC GCC GCG ATC GCC ATG
pCMV6 Insert End Rev	5'GCC GGC TTA AAC CTT ATC GTC GTC
T7 Forward	5'TAA TAC GAC TCA CTA TAG GG
CMV Forward	5'CGC AAA TGG GCG GTA GGC GTG
hEF2 Seq Primer Reverse	5'GAT GGC TGG CAA CTA GAA GGC AC
BGH Reverse	5'TAG AAG GCA CAG TCG AGG
hFUR SDS>ADA F [BM]	5'ACG CAG AAG AGG ACG AGG GCC G
hFUR SDS>ADA R [BM]	5'CAG CCG GCC ACT CCT CCT GCC A
hFUR SDS>DDD F [BDG]	5'ACG ATG AAG AGG ACG AGG GCC G
hFurin CDNA Truncated R	5'TGG CAT CGT ACG CGT GGC CTG ACT GGA CGT GAG
hFUR Exon 1 3 PCR For	5'CAT CTG TCC ATC TAT GTG GCT GGC
hFUR Exon 1 3 PCR Rev	5'GCA GGA GGA AAG GAA ACG AAT CAG AG
hFur Exon 1 Seq	5'GCA GGG CAG CTT TAG TGC GTA G
pUC57 Furin Ex 2 OvEx C+A	5'CCG CAT CAC CAT CAT CAC CAC TAA GAA TTC ATC GCA GGG CAG CTT TAG TGC GTA G
pUC57 Furin Ex 2 OvEx D+B	5'GCG GAT AAC AAT TTC ACA CAG GAA ACA GCT ATG ACC TCA CTC TCA CTG GGA TAT GCT GC
hFurin cDNA Catalytic Domain Sequencing F	5'GTG GCA AAG CGA CGG ACT AAA C
hFurin cDNA P Domain R primer	5'GAT GTC GAT GAT GCA CTT CCG C
hFurin cDNA P Domain F	5'GCG GAA GTG CAT CAT CGA CAT C
hFurin cDNA Catalytic Domain R	5'GTT TAG TCC GTC GCT TTG CCA C
M13 R	5'CAG GAA ACA GCT ATG AC

Biology Open • Supplementary information

Table S2. STR profiles

Locus	HEK293†	HEK293 FRT	ΔFUR293
Amelogenin	X[2–11]	X	X
CSF1PO	12*[10]<br>11,12[4–9,11]	12	12
D1S1656	*Not reported*	15,17.3	15,17.3
D2S441	*Not reported*	11,15	11,15
D2S1338	19[8]	19	19
D3S1358	15*[2]<br>15,17[8,9]	15,17	15,17
D5S818	8*[10]<br>8,9[2,4–9,11]	8	8
D7S820	11,12[2,4–11]	11,12	11,12
D8S1179	12,14[2,3,8]	12	12,14
D10S1248	*Not reported*	14	14
D12S391	*Not reported*	19,21	19,21
D13S317	12[4]<br>12,14[2,5–11]	12,14	12,14
D16S539	9<br>9,13[4–8,10,11]	9,13	9,13
D18S51	17[2,3]<br>17,18[3]<br>18[8]	17,18	17,18
D19S433	15,18<br>18[8]	15,18	18
D21S11	28,30.2[2,3,8]	28,30.2	28,30.2
D22S1045	*Not reported*	16	16
FGA	23[2,3,8,9]	23	23
Penta D	9,10	*Not evaluated*	*Not evaluated*
Penta E	7,15	*Not evaluated*	*Not evaluated*
SE33	*Not reported*	17	17
TH01	7,9.3[3–11]	7,9.3	7,9.3
TPOX	11[4–11]	11	11
vWA	16*[3]<br>16,19[2–11]	16,19	16,19

†STR data as reported for HEK293 (RRID:CVCL_0045) by the *Cellosaurus* database[12]. Note that multiple variants were reported for several loci. Additional variants not listed in the *Cellosaurus* database (indicated by *) were also noted in the literature and in biological resource centers. Not all loci could be confirmed, but references are provided where possible.

Table S3. Statistical analysis of normalized EC50 differences between furin transgenic cell lines

	ΔFur293	Fur #1	Fur #2	Fur^{ADA} #1	Fur^{ADA} #2	Fur^{DDD} #1	Fur^{DDD} #2	$Fur^{Ala\text{-}295}$ #1	$Fur^{Ala\text{-}295}$ #2
ΔFur293									
Fur #1	0.0150								
Fur #2	0.0152	0.1423							
Fur^{ADA} #1	0.0134	0.0082	0.0052						
Fur^{ADA} #2	0.0126	0.0322	0.0240	0.8619					
Fur^{DDD} #1	0.0284	0.0129	0.0137	0.0035	0.0138				
Fur^{DDD} #2	0.0140	0.0064	0.0010	0.0029	0.0192	0.3624			
$Fur^{Ala\text{-}295}$ #1	0.0395	0.0383	0.0291	0.2877	0.2994	0.0201	0.0232		
$Fur^{Ala\text{-}295}$ #2	0.0129	0.0011	0.0004	0.5947	0.6885	0.0003	0.0003	0.9999	

Biology Open • Supplementary information

References

(1) *Benchling [Biology Software]. (2019). Retrieved from https://Benchling.Com.*

(2) Bylund, L.; Kytölä, S.; Lui, W.-O.; Larsson, C.; Weber, G. Analysis of the Cytogenetic Stability of the Human Embryonal Kidney Cell Line 293 by Cytogenetic and STR Profiling Approaches. *Cytogenet Genome Res* 2004, *106* (1), 28–32. https://doi.org/10.1159/000078556.

(3) Masters, J. R.; Thomson, J. A.; Daly-Burns, B.; Reid, Y. A.; Dirks, W. G.; Packer, P.; Toji, L. H.; Ohno, T.; Tanabe, H.; Arlett, C. F.; Kelland, L. R.; Harrison, M.; Virmani, A.; Ward, T. H.; Ayres, K. L.; Debenham, P. G. Short Tandem Repeat Profiling Provides an International Reference Standard for Human Cell Lines. *Proc Natl Acad Sci U S A* **2001**, *98* (14), 8012–8017. https://doi.org/10.1073/pnas.121616198.

(4) *Cell Resource Center for Biomedical Research, Tohoku University: TKG 0306 ： ： 293*. http://www2.idac.tohoku.ac.jp/dep/ccr/TKGdate/TKGvo103/0306.html (accessed 2021-04-12).

(5) *American Type Culture Collection (ATCC): 293 [HEK-293] ATCC® CRL-1573™*. https://www.atcc.org/Products/All/CRL-1573.aspx#specifications (accessed 2021-04-09).

(6) *Japanese Collection of Research Bioresources Cell Bank (JCRB): JCRB9068 293*. https://cellbank.nibiohn.go.jp/~cellbank/en/search_res_det.cgi?RNO=JCRB9068 (accessed 2021-04-09).

(7) *European Collection of Authenticated Cell Cultures (ECACC): ECACC General Cell Collection: 293*. https://www.phe-culturecollections.org.uk/products/celllines/generalcell/detail.jsp?refId=85120602&collection=ecacc_gc (accessed 2021-04-12).

(8) *Cell Lines Service GmbH (CLS): HEK293*. https://www.clsgmbh.de/p777_HEK293.html (accessed 2021-04-09).

(9) *Korean Cell Line Bank (KCLB): KCLB #21573 293[HEK-293]*. https://cellbank.snu.ac.kr/english/sub/catalog.php?s_cellid=578&page=detail_info&CatNo=59&strQ=21573 (accessed 2021-04-12).

(10) *Bioresource Collection and Research Center (BCRC): BCRC Number 60019: 293*. https://catalog.bcrc.firdi.org.tw/BcrcContent?bid=60019 (accessed 2021-04-12).

(11) *Sigma-Aldrich 293 Cell Line human CB_85120602*. Sigma-Aldrich. https://www.sigmaaldrich.com/catalog/product/sigma/cb_85120602 (accessed 2021-04-20).

(12) Bairoch, A. The Cellosaurus, a Cell-Line Knowledge Resource. *J Biomol Tech* **2018**, *29* (2), 25–38. https://doi.org/10.7171/jbt.18-2902-002.

Biology Open • Supplementary information
